# Supplementary material for: Synthesis and Biological Activity of a VHL-Based PROTAC Specific for p38α
Source: Cancers (Basel). 2023 Jan 18;15(3):611. doi: 10.3390/cancers15030611 (PMC9913880; doi:10.3390/cancers15030611)
Supplement: Supplementary file 1 [file cancers-15-00611-s001.zip › cancers-2144579-supplementary.pdf]

Supplementary Materials

Synthesis and biological activity of a VHL-based PROTAC specific for p38α

Mónica Cubillos-Rojas[1,†], Guillem Loren[1,†], Yusuf Z. Hakim[1], Xavier Verdaguer[1,2], Antoni Riera[1,2,*] and Angel R. Nebreda[1,3,*]

[1]Institute for Research in Biomedicine (IRB Barcelona), The Barcelona Institute of Science and Technology, Baldiri Reixac 10, 08028 Barcelona, Spain

[2]Dept. Química Inorgànica i Orgànica, Universitat de Barcelona, Martí i Franquès 1, 08028 Barcelona, Spain.

[3]ICREA, Pg. Lluís Companys 23, 08010 Barcelona, Spain

Table of Contents

Figure S1. ^{1}H and ^{13}C NMR spectra and HPLC chromatograms of **NR-11a**..............2

Figure S2. ^{1}H and ^{13}C NMR spectra and HPLC chromatograms of **NR-11b** 4

Figure S3. ^{1}H and ^{13}C NMR spectra and HPLC chromatograms of **NR-11c**............ 6

Figure S4. HPLC chromatograms of **NR-11c***... 8

File S1. Uncropped blots …………………………………………………………..9

Figure S1. ^{1}H and ^{13}C NMR spectra and HPLC chromatograms of **NR-11a.**

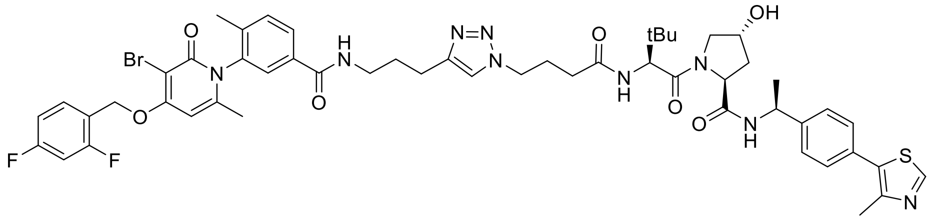

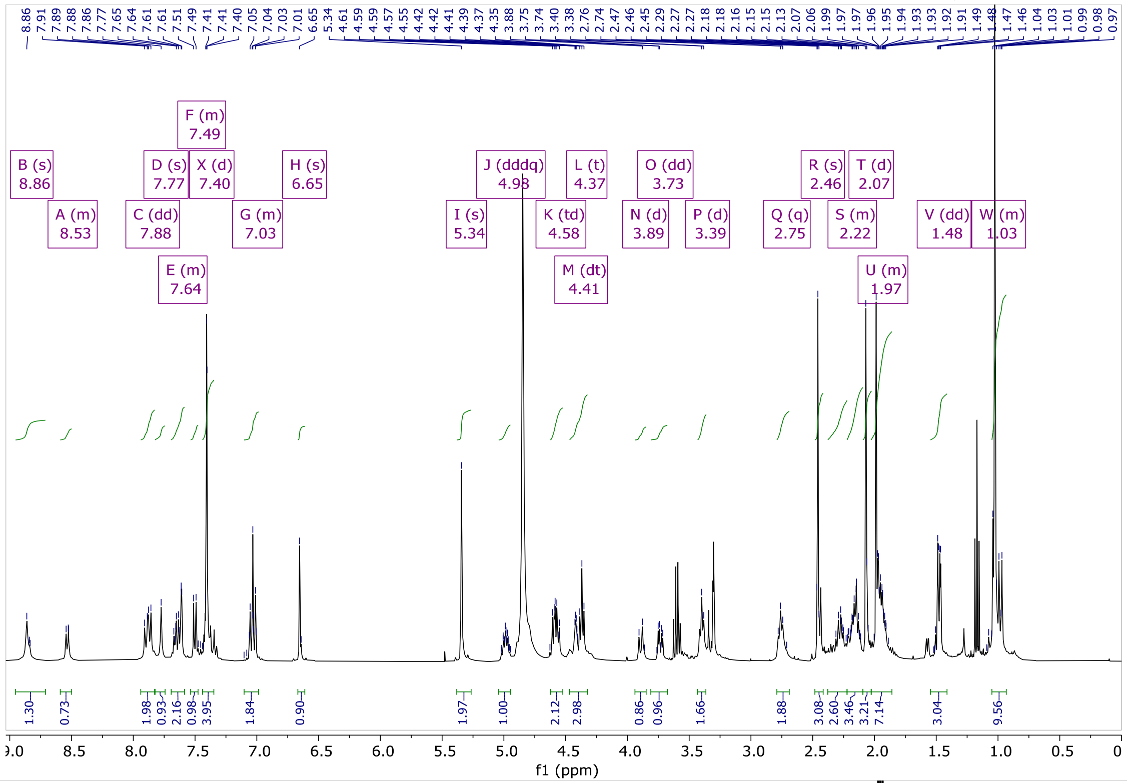

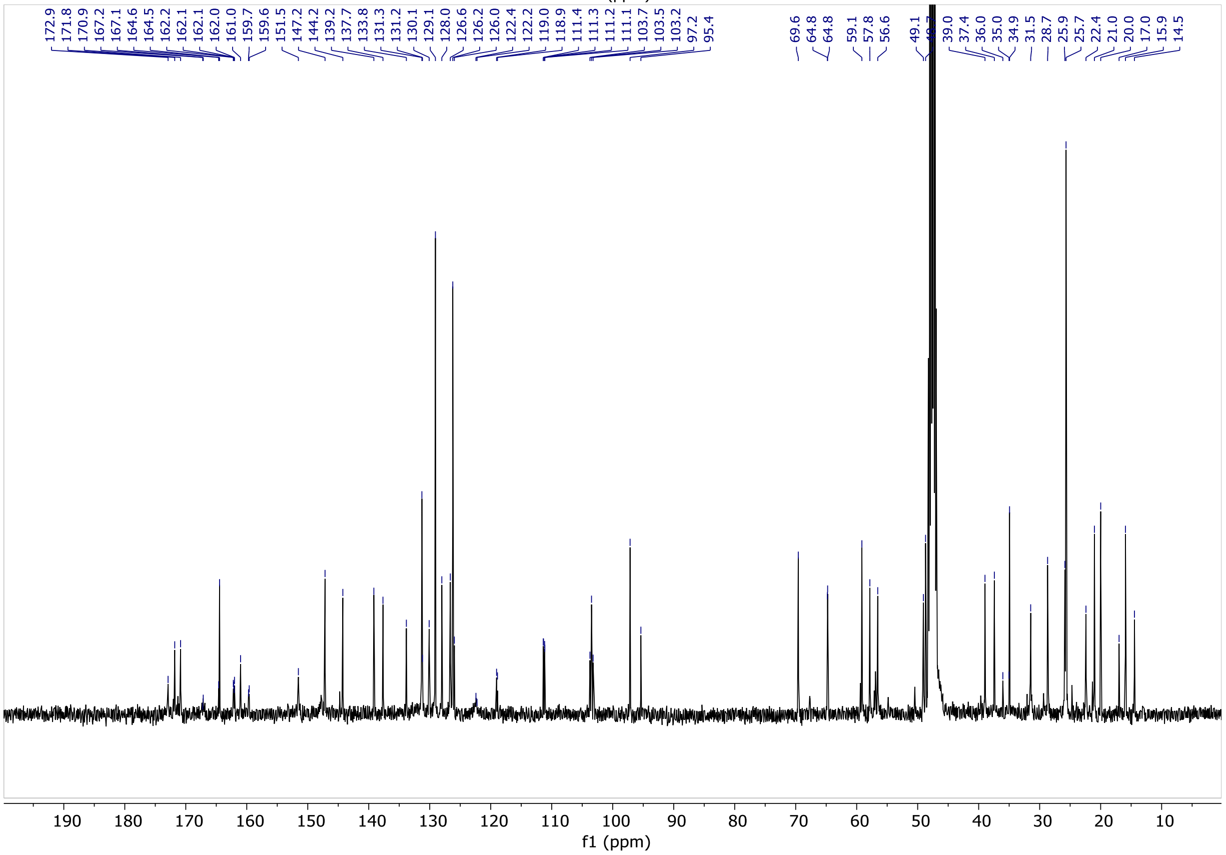

```
                                                                  1.5   ml/min
 Injection date:  11/18/2020     9:25:57 PM                         1.0uL
     Sample Name: YZH20                                           58
   Acq Operator:  Analyst                                           282bar
Analysis method:  C:\Chem32\1\Methods\1W_4_KN50_01_GD95B10F1-5S11_T40P.M

     Method Info  Kinetex EVO C18 50x 4.6mm, 2.6um; Mobile phase: 10mM
                  NH4HCO3 pH8  / ACN (95:5)--0.5min---(95:5)---6.5min----(0:
                  100)---2min----(0:100) post run 1.5 min
     Sample Info: Aprox. 1 mg/mL ACN
                  Walkup method: 'M1_Positive_Standard'
                  Target:
```

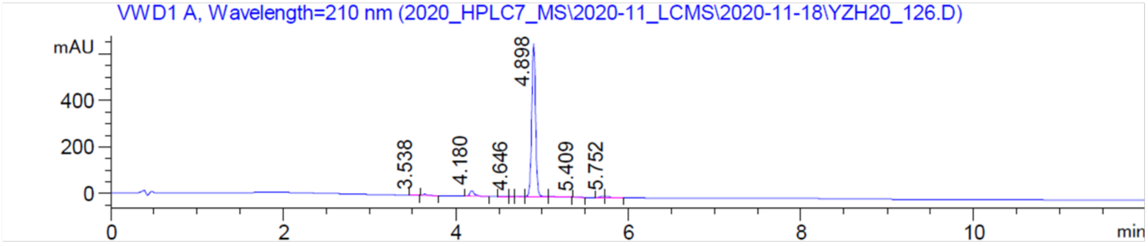

#	Meas. Ret	Peak	Width	Area	Response
1	3.538	MM	0.066	2.996	0.134
2	3.633	VB	0.044	17.127	0.765
3	4.180	VB	0.052	85.401	3.814
4	4.561	BV	0.054	5.074	0.227
5	4.646	VV	0.049	4.556	0.204
6	4.737	VV	0.074	12.600	0.563
7	4.898	VV	0.048	2.057e3	91.890
8	5.115	VB	0.074	12.315	0.550
9	5.409	BB	0.053	3.790	0.169
#	5.694	VV	0.051	15.852	0.708
#	5.752	VB	0.056	21.858	0.976

Figure S2. ^{1}H and ^{13}C NMR spectra and HPLC chromatograms of **NR-11b.**

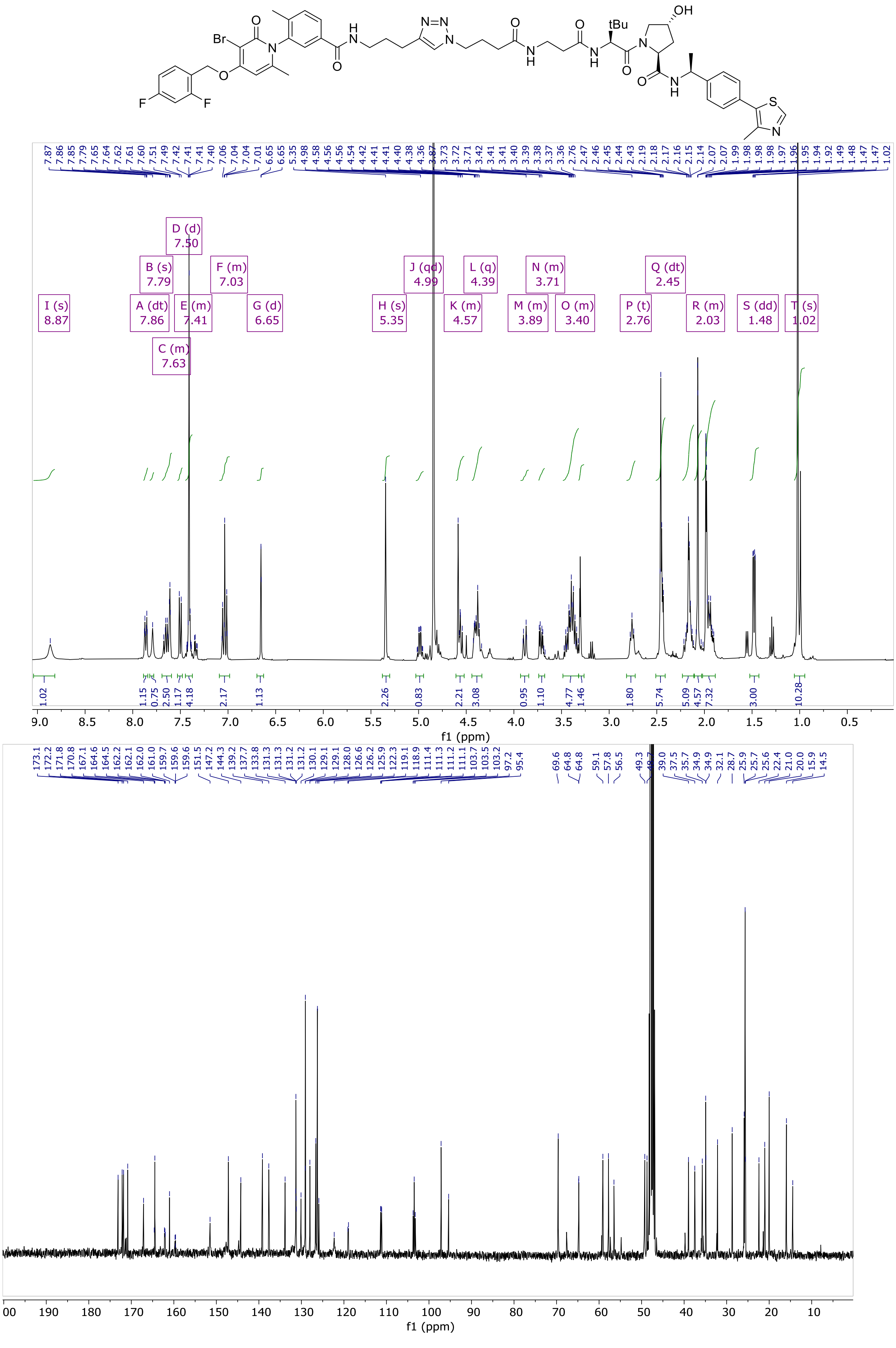

1.5 ml/min

Injection date: 11/18/2020 9:10:44 PM 1.0uL

Sample Name: YZH19 57

Acq Operator: Analyst 281bar

Analysis method: C:\Chem32\1\Methods\1W_4_KN50_01_GD95B10F1-5S11_T40P.M

Method Info Kinetex EVO C18 50x 4.6mm, 2.6um; Mobile phase: 10mM NH4HCO3 pH8 / ACN (95:5)--0.5min---(95:5)---6.5min----(0:100)---2min----(0:100) post run 1.5 min

Sample Info: Aprox. 1 mg/mL ACN

Walkup method: 'M1_Positive_Standard'

Target:

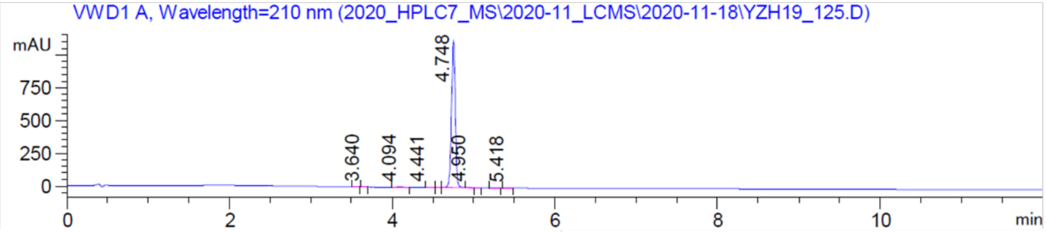

#	Meas. Ret	Peak	Width	Area	Response
1	3.543	MM	0.056	2.147	0.059
2	3.640	MM	0.044	2.193	0.061
3	4.094	BB	0.057	24.734	0.683
4	4.441	MF	0.073	1.724	0.048
5	4.572	MF	0.056	5.836	0.161
6	4.748	FM R	0.053	3.570e3	98.567
7	4.950	MM T	0.064	2.101	0.058
8	5.243	BB	0.047	5.987	0.165
9	5.418	BV	0.053	7.190	0.198

Figure S3. ^{1}H and ^{13}C NMR spectra and HPLC chromatograms of **NR-11c.**

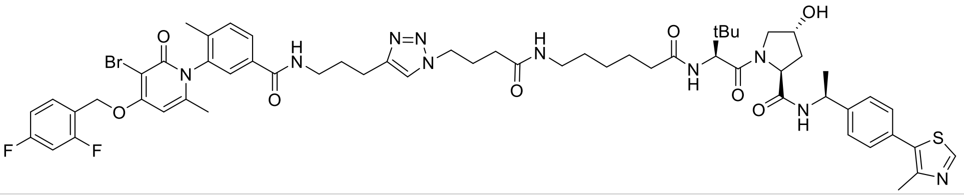

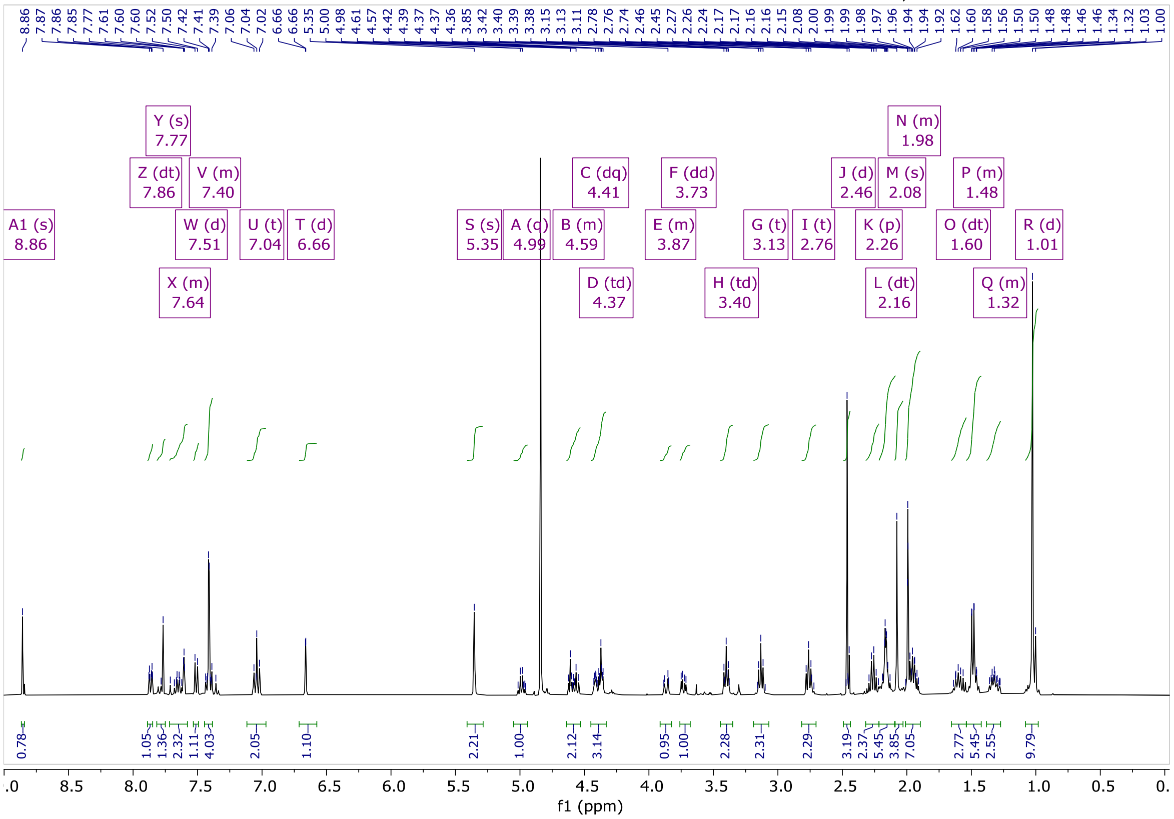

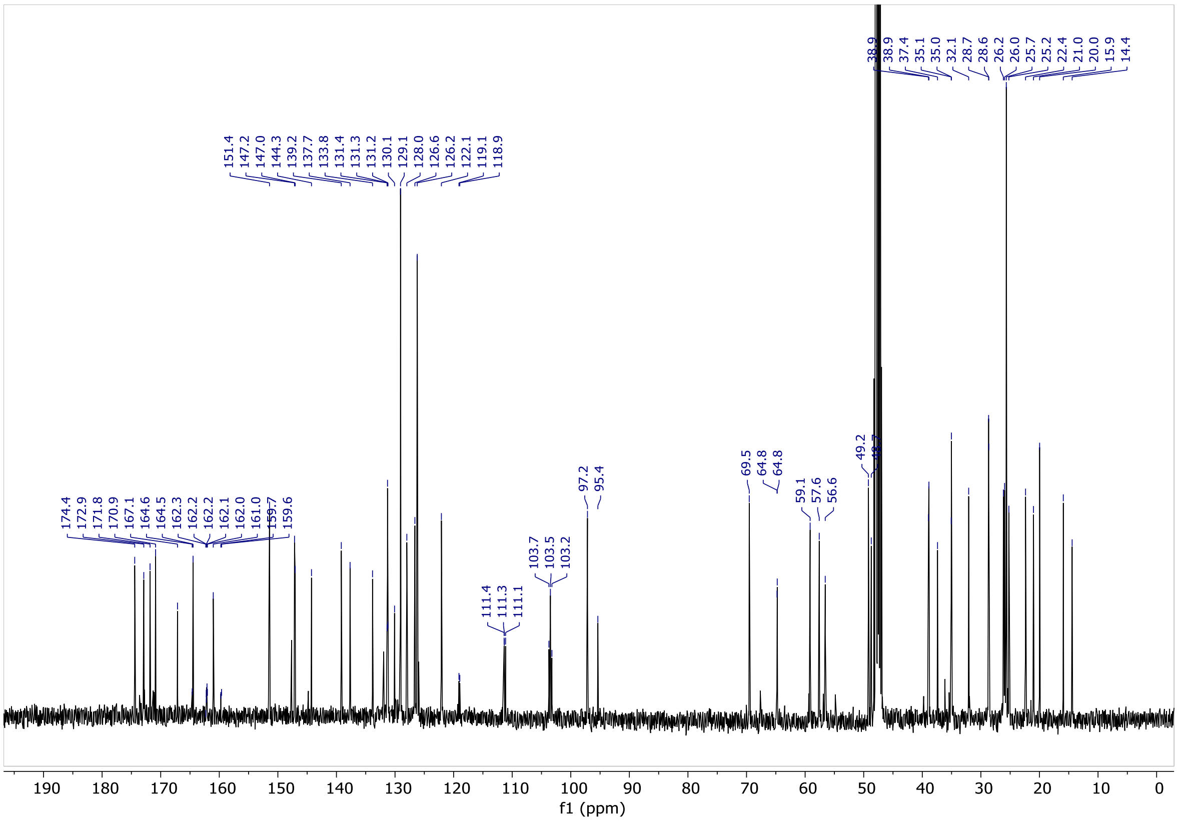

1.5 ml/min

Injection date: 12/4/2020 7:51:21 PM 1.0uL

Sample Name: YZH028 81

Acq Operator: Analyst 225bar

Analysis method: C:\Chem32\1\Methods\1W_4_KN50_01_GD95B10F1-5S11_T40P.M

Method Info Kinetex EVO C18 50x 4.6mm, 2.6um; Mobile phase: 10mM NH4HCO3 pH8 / ACN (95:5)--0.5min---(95:5)---6.5min----(0:100)---2min----(0:100) post run 1.5 min

Sample Info: Aprox. 1 mg/mL MeOH

Walkup method: 'M1_Positive_Standard'

Target:

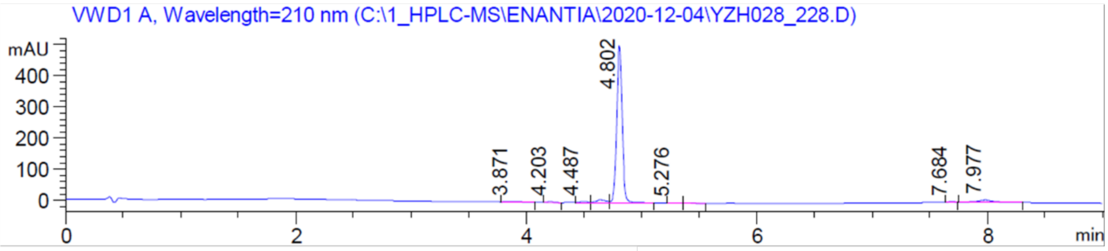

#	Meas. Ret	Peak	Width	Area	Response
1	3.871	BB	0.095	17.074	0.925
2	4.203	VB	0.052	9.677	0.524
3	4.487	BV	0.064	15.761	0.854
4	4.635	VV	0.082	54.632	2.961
5	4.802	VB	0.050	1.663e3	90.121
6	5.276	BV	0.054	3.558	0.193
7	5.415	VB	0.055	7.088	0.384
8	7.684	MM	0.052	4.341	0.235
9	7.977	MF	0.162	70.140	3.802

Figure S4. HPLC chromatograms of **NR-11c*.**

```
Datafile:C:\1\DATA\2021_HPLC7-MS\2111_HPLC7-MS\2021-11-03\GL796_27.D

 Injection date:  03-Nov-21  11:31:45     Flow:  1.5ml/min          1.0uL
     Sample Name: GL796                   Pos:   27                 351bar
 Analysis method:  C:\1\Methods\1W_4_KN50_01_GD95B_L210F1-5S11_T40P.M

 Kinetex EVO C18 50x 4.6mm, 2.6um; Mobile phase: 10mM NH4HCO3 pH8  / ACN (
 95:5)--0.5min---(95:5)---6.5min----(0:100)---2min----(0:100) post run 1.5
 min; Tª40°C

 Aprox. 1 mg/mL MeOH
 Walkup method: 'M1_Positive_Standard_9min'
 Target:
```

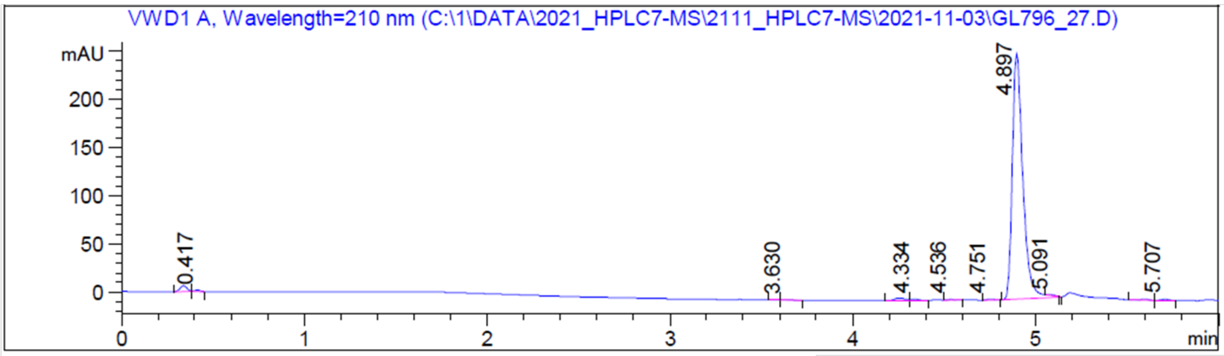

Signal: 2->   : VWD1 A, Wavelength=210 nm

#	Meas. Ret. Time	Area	Area %
1	0.338	15.26	1.44
2	0.417	3.14	0.30
3	3.572	1.17	0.11
4	3.630	1.26	0.12
5	4.254	10.62	1.00
6	4.334	4.83	0.46
7	4.536	2.30	0.22
8	4.751	2.56	0.24
9	4.897	1009.97	95.14
10	5.091	2.71	0.26
11	5.601	4.34	0.41
12	5.707	3.36	0.32

Figure 2

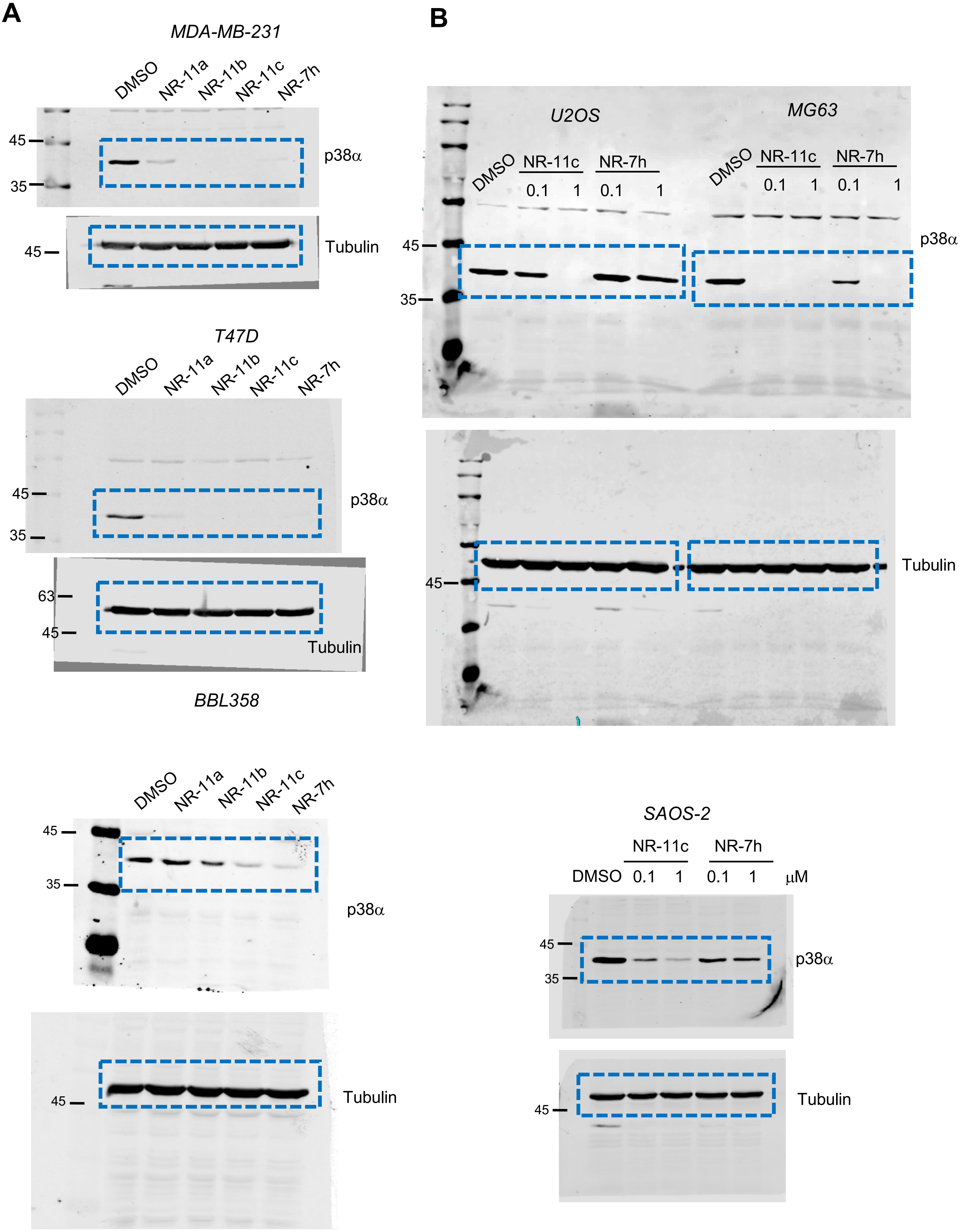
A
MDA-MB-231
DMSO
NR-11a
NR-11b
NR-11c
NR-7h
45
35
p38α
45
Tubulin
T47D
DMSO
NR-11a
NR-11b
NR-11c
NR-7h
45
35
p38α
63
45
Tubulin
BBL358
DMSO
NR-11a
NR-11b
NR-11c
NR-7h
45
35
p38α
45
Tubulin
B
U2OS
MG63
DMSO
NR-11c
NR-7h
0.1
1
0.1
1
DMSO
NR-11c
NR-7h
0.1
1
0.1
1
45
35
p38α
45
Tubulin
SAOS-2
NR-11c
NR-7h
DMSO
0.1
1
0.1
1
µM
45
35
p38α
45
Tubulin

Figure 3

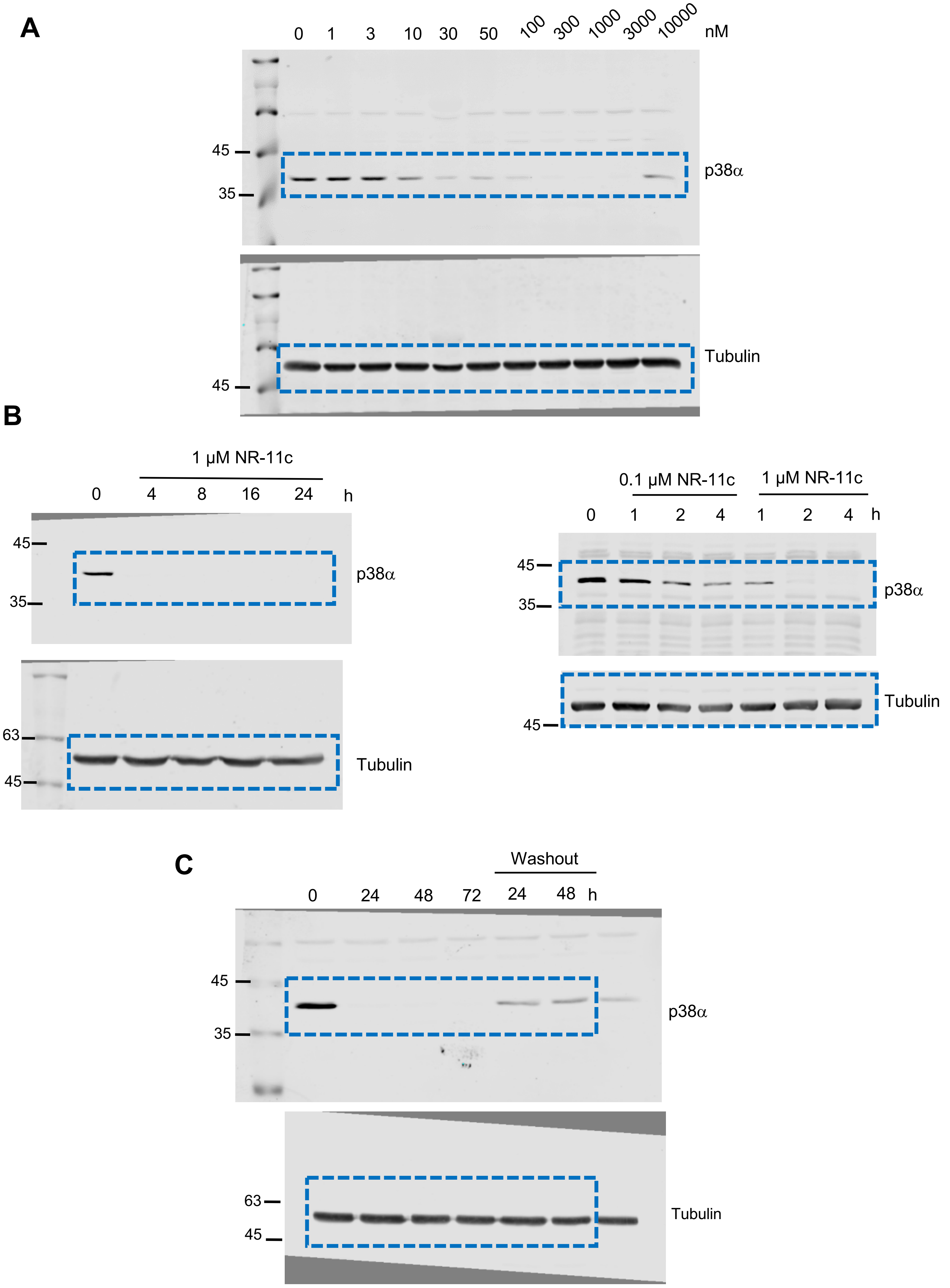
A
0 1 3 10 30 50 100 300 1000 3000 10000 nM
45
35
p38α
Tubulin
45
B
1 μM NR-11c
0 4 8 16 24 h
45
35
p38α
63
45
Tubulin
0.1 μM NR-11c
1 μM NR-11c
0 1 2 4 1 2 4 h
45
35
p38α
Tubulin
45
C
Washout
0 24 48 72 24 48 h
45
35
p38α
63
45
Tubulin

Figure 4

A

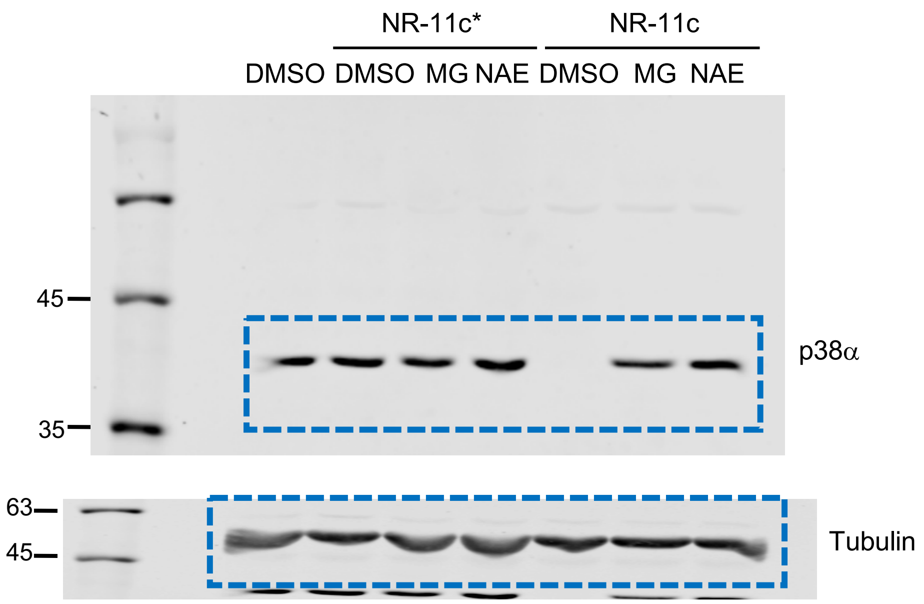

NR-11c*
NR-11c
DMSO DMSO MG NAE DMSO MG NAE
45
35
p38α
63
45
Tubulin

C

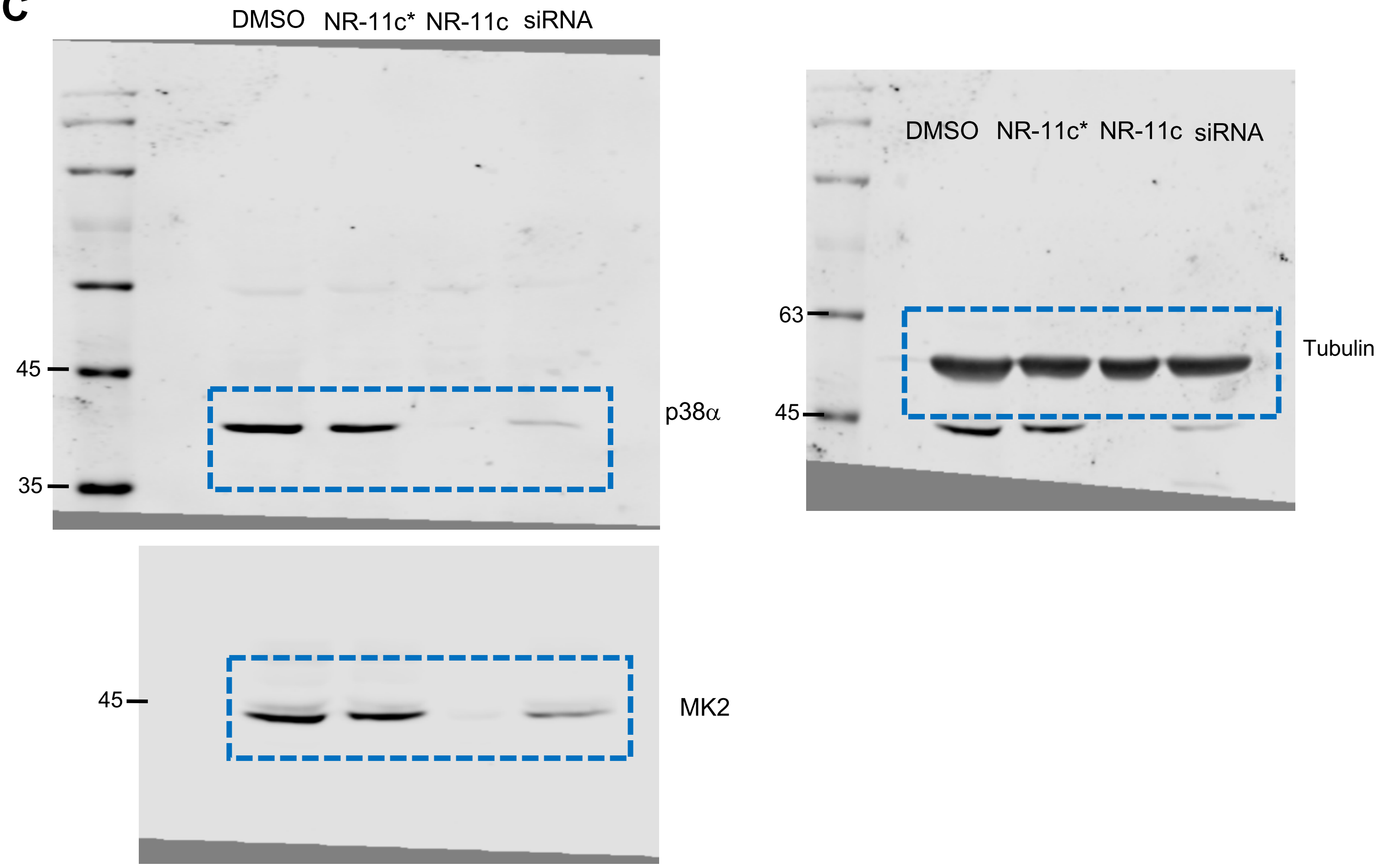

DMSO NR-11c* NR-11c siRNA
45
35
p38α
45
MK2
DMSO NR-11c* NR-11c siRNA
63
45
Tubulin

Figure 5

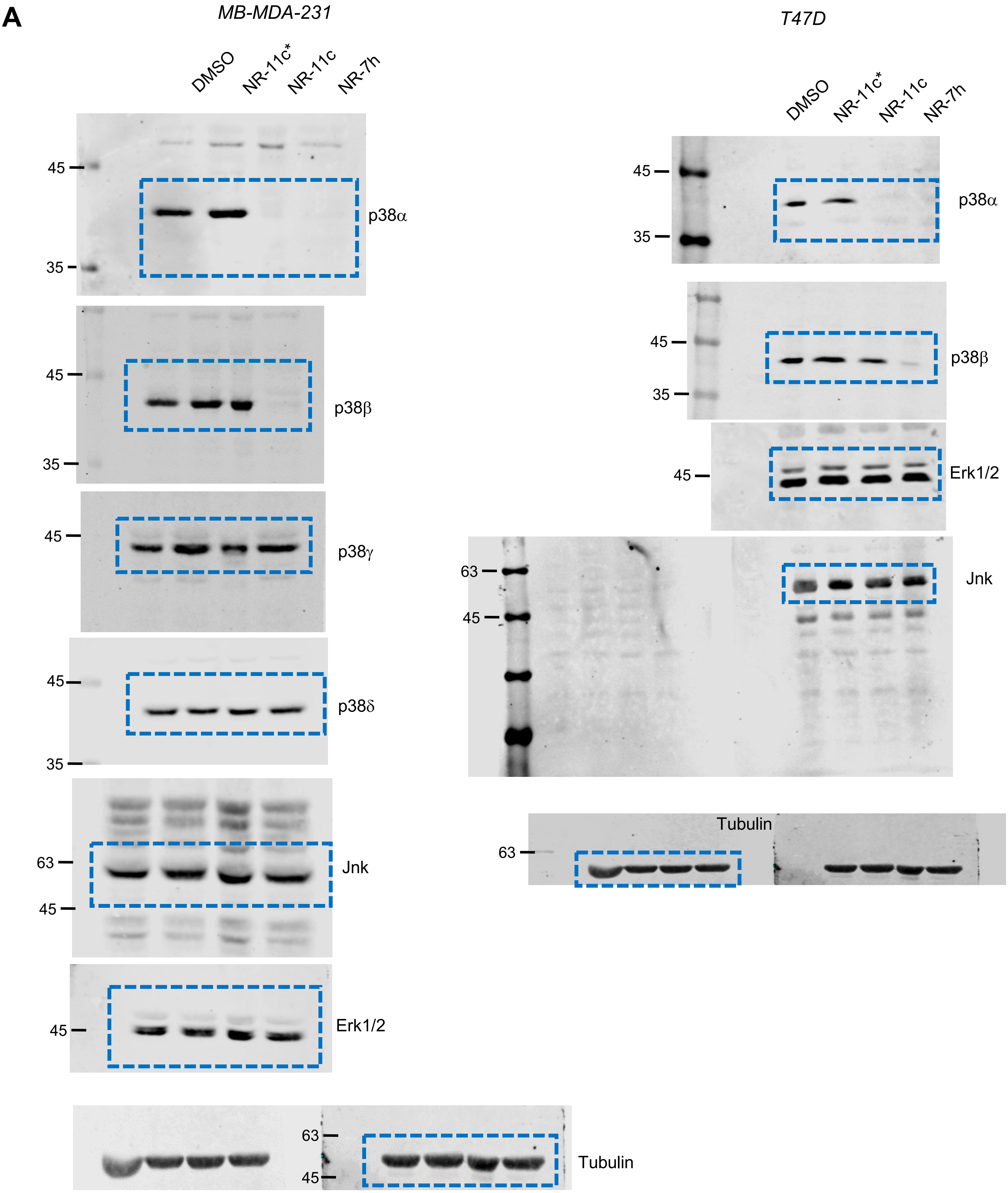
A
MB-MDA-231
DMSO
NR-11c*
NR-11c
NR-7h
45
35
p38α
p38β
p38γ
p38δ
63
Jnk
Erk1/2
Tubulin
T47D

Figure 6

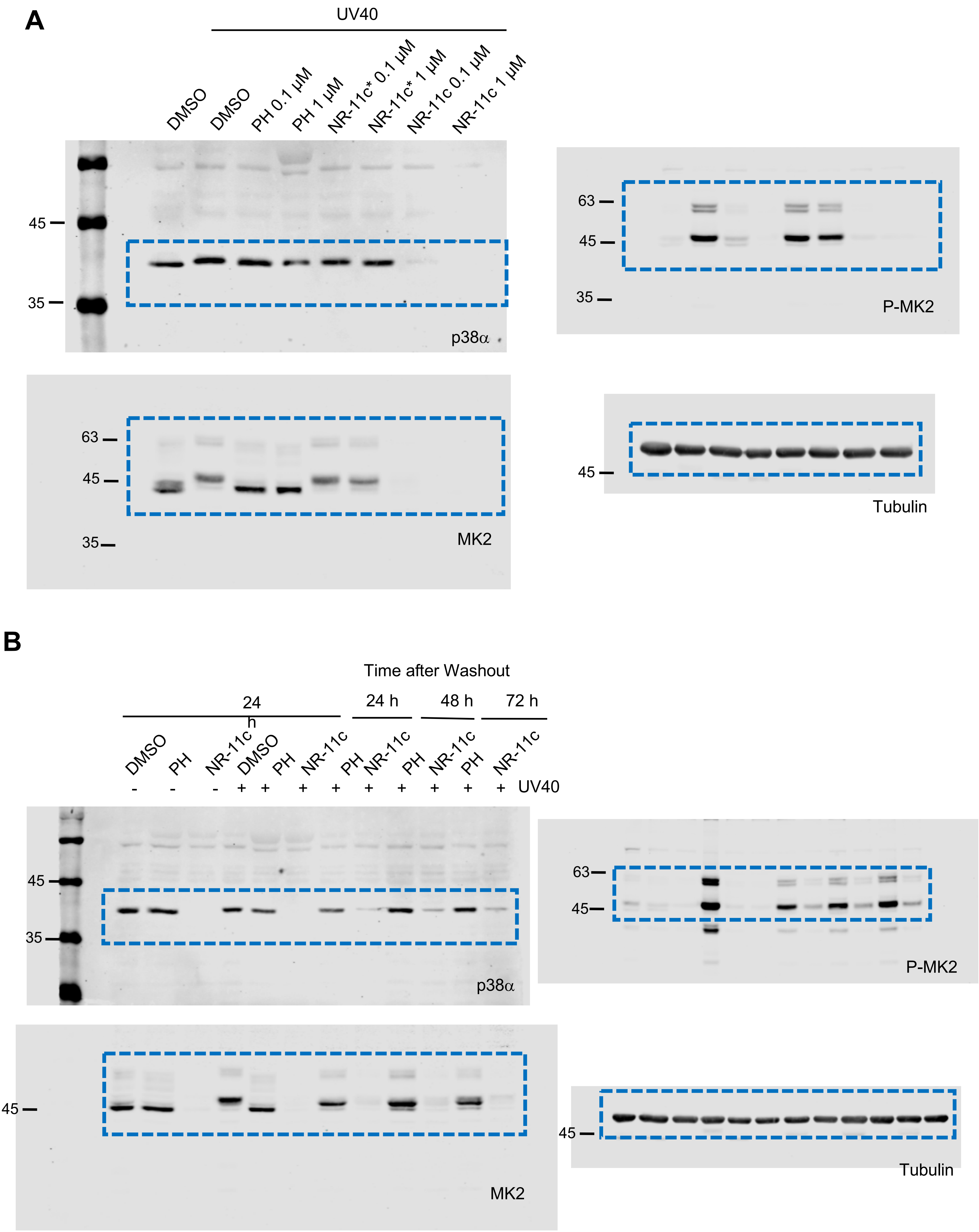
A
UV40
DMSO
DMSO
PH 0.1 μM
PH 1 μM
NR-11c* 0.1 μM
NR-11c* 1 μM
NR-11c 0.1 μM
NR-11c 1 μM
45
35
p38α
63
45
35
P-MK2
63
45
35
MK2
45
Tubulin
B
Time after Washout
24 h
24 h
48 h
72 h
DMSO
PH
NR-11c
DMSO
PH
NR-11c
PH
NR-11c
PH
NR-11c
PH
NR-11c
- - - + + + + + + + + +
UV40
45
35
p38α
63
45
P-MK2
45
MK2
45
Tubulin

Figure 7

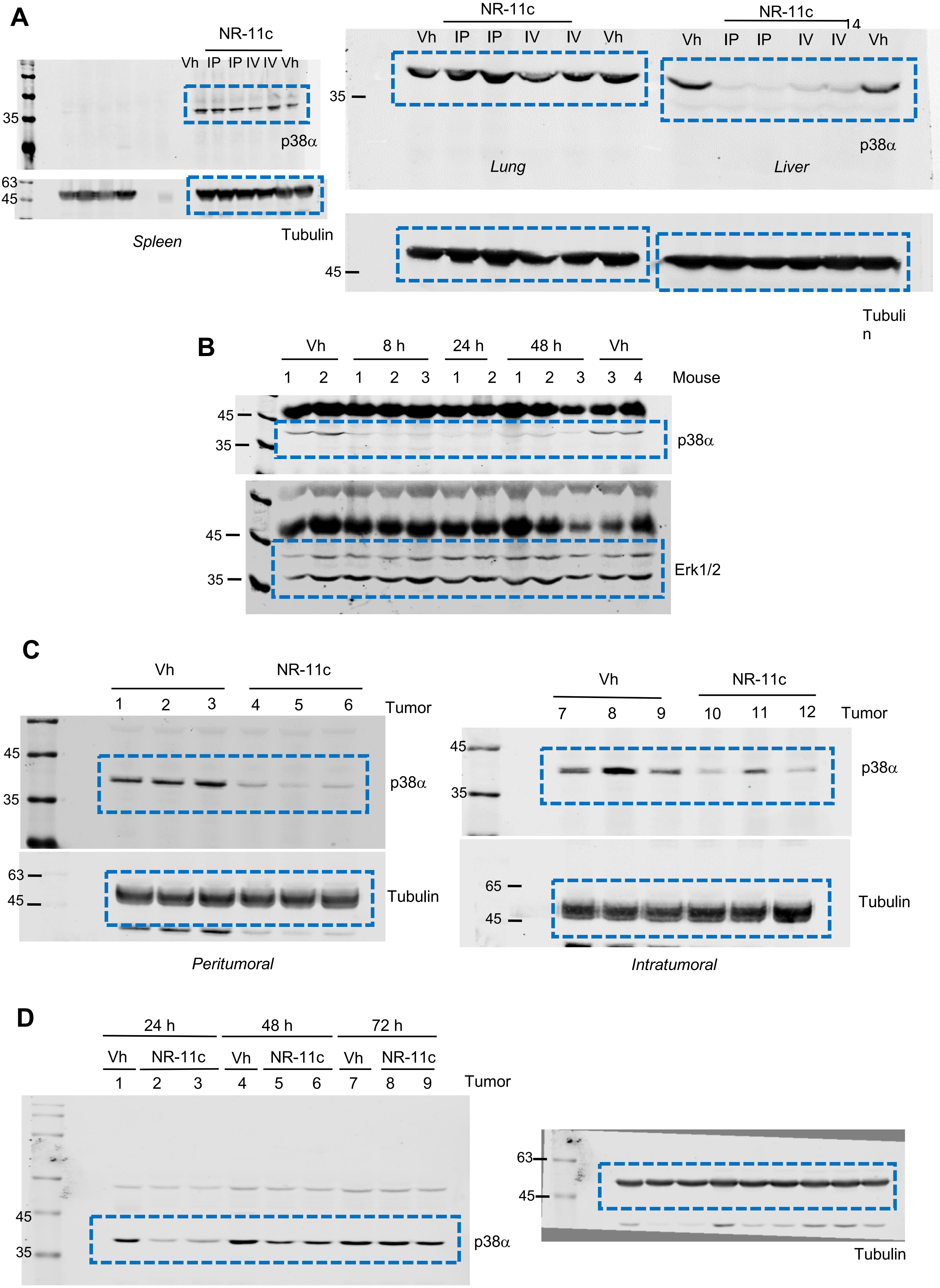
A
NR-11c
Vh IP IP IV IV Vh
35
p38α
63
45
Tubulin
Spleen
NR-11c
Vh IP IP IV IV Vh
35
Lung
Liver
p38α
45
Tubuli
n
14
B
Vh 8 h 24 h 48 h Vh
1 2 1 2 3 1 2 1 2 3 3 4
Mouse
45
35
p38α
45
35
Erk1/2
C
Vh NR-11c
1 2 3 4 5 6
Tumor
45
35
p38α
63
45
Tubulin
Peritumoral
Vh NR-11c
7 8 9 10 11 12
Tumor
45
35
p38α
65
45
Tubulin
Intratumoral
D
24 h 48 h 72 h
Vh NR-11c Vh NR-11c Vh NR-11c
1 2 3 4 5 6 7 8 9
Tumor
45
35
p38α
63
45
Tubulin
